# PHIDIAS: a pathogen-host interaction data integration and analysis system

**DOI:** 10.1186/gb-2007-8-7-r150

**Published:** 2007-07-30

**Authors:** Zuoshuang Xiang, Yuying Tian, Yongqun He

**Affiliations:** 1 Unit for Laboratory Animal Medicine, University of Michigan, 1150 W. Medical Dr., Ann Arbor, MI 48109, USA; 2 Department of Microbiology and Immunology, University of Michigan, 1150 W. Medical Dr., Ann Arbor, MI 48109, USA; 3 Center for Computational Medicine and Biology, University of Michigan, 100 Washtenaw Ave, Ann Arbor, MI 48109, USA; 4 Medical School Information Services, University of Michigan, 535 W. William St., Ann Arbor, MI, USA

## Abstract

The Pathogen-Host Interaction Data Integration and Analysis System (PHIDIAS) is a web-based database system that serves as a centralized source to search, compare, and analyze integrated genome sequences, conserved domains, and gene expression data related to pathogen-host interactions (PHIs) for pathogen species designated as high priority agents for public health and biological security. In addition, PHIDIAS allows submission, search and analysis of PHI genes and molecular networks curated from peer-reviewed literature. PHIDIAS is publicly available at .

## Abstract 2

PHIDIAS is a web-based database system serving as a centralized source to search, compare and analyse integrated genome sequences, conserved domains and transcriptional data related to pathogen-host interactions.

## Rationale

An infectious disease is the result of an interactive relationship between a pathogen and its host. According to estimations of the World Health Organization, infectious diseases caused 14.7 million deaths in 2001, accounting for 26% of the total global mortality [1]. Integration and analysis of various data related to pathogens and pathogen-host interactions (PHIs) will yield a better understanding of, and means for, the control of infectious diseases induced by such pathogens.

Completely sequenced genomic information provides valuable information for gene and protein functions, and intra-organismic processes. Pathogen genome information also lays a foundation for the study of the interactions between host and microbial organisms. Several genome data resources, such as the National Center for Biotechnology Information (NCBI), European Bioinformatics Institute (EBI) and Swiss Institute of Bioinformatics (SIB), are available to the public. However, data obtained from these sources often are not integrated. Lack of such integration prompted us to develop the *Brucella *Bioinformatics Portal (BBP) [2]. This program allows integration of data from more than 20 sources including information on the *Brucella *genome. The same strategy can be expanded to include other pathogens, thereby enhancing our ability to conduct comparative studies. The program can be modified to include additional features not yet available in BBP. For example, protein conserved domains (distinct units of molecular evolution usually associated with particular molecular functions) could be listed. The NCBI Conserved Domain Database (CDD) mirrors several collections, including the Protein families database of alignments (Pfam) [3], Simple Modular Architecture Research Tool (SMART) [4], and Clusters of Orthologous Groups (COG) [5], and thus provides comprehensive information about conserved protein domains. Conserved domains are critical for protein functions and provide important clues about microbial pathogenesis and interactions between pathogens and hosts.

While CDD contains conserved domains derived from various eukaryotic and prokaryotic organisms [6], it is difficult to compare and analyze pathogen-specific conserved domains. The availability of a program that permits the acquisition and storage of pathogen-specific domain information in an integrated system would be extremely useful, as would the combination of such a database with BLAST search programs and other programs for the determination of sequence analyses. To facilitate comparison and better understanding of pathogens and fundamental PHI mechanisms, it is necessary to integrate genome information from publicly important pathogens with effective tools for browsing, searching, and analyzing annotated genome sequences and conserved domains. Such an integrated system would also benefit from the inclusion of large amounts of published literature data relating to pathogens and their interactions with host immune systems. To allow machine-readable data exchange of the now voluminous pathogen information, He *et al*. [7] developed an Extensible Markup Language (XML)-based Pathogen Information Markup Language (PIML). PIML contains comprehensive pathogen-oriented information, including pathogen taxonomy, genomic information, life cycle, epidemiology, induced diseases in host, diagnosis, treatment, and relevant laboratory analysis. A list of PIML documents addressing pathogens deemed of high priority for public health and biological defense have been created and are available on the worldwide web or through a web service [7]. However, compared to relational databases, XML databases do not efficiently support query functions and scalability. These deficiencies prompted us to design a web-based relational database system to store and query PIML data. The database system can also integrate efficiently other PHI-related data, including manually curated information related to the pathobiology and management of laboratory animals that are given high priority pathogens [8].

The molecular functions of pathogen and host genes as well as their roles in specific PHI pathways have been extensively studied. Molecules that play important roles in the virulence of pathogens and in the host immune defense are particularly important for PHI. A systematic collation from the literature of these molecules and their functions is lacking. Once PHI-related molecules are collated, the next step is to illustrate molecular interactions and pathways involving these molecules. Existing pathway databases, such as the Kyoto Encyclopedia of Genes and Genomes (KEGG) [9], BioCyc [10,11], and Biomolecular Interaction Network Database (BIND) [12], contain pathways for various metabolic and molecular interactions of different organisms. Although richly documented, the networks of microbial and host molecular and cellular interactions that occur during pathogenic infections of hosts are underrepresented in current database systems. He and colleagues [13] developed the Molecular Interaction Network Markup Language (MINetML, previously called ProNetML) to summarize information related to microbial pathogenesis. However, MINetML cannot be exchanged with other standard data exchange formats such as the Biological Pathways Exchange format (BioPAX) [14]. This deficiency prevents active data exchange and communication with biological pathway databases. In addition, there is no effective MINetML visualization tool available.

Experimental methodologies, including microarrays and mass spectrometry, provide abundant sources of gene expression data. Publicly available gene expression data repositories, including the NCBI Gene Expression Omnibus (GEO) [15] and the EBI ArrayExpress [16] store large amounts of gene expression data, much of which is related to interactions between pathogens and hosts. Summaries of gene expression experiments and gene profiles allow querying and comparison of PHI-related gene expression patterns.

To better understand the intricate interactions between pathogens and hosts, we have now developed a web-based PHI data integration and analysis system (PHIDIAS) that permits integration and analysis of genome sequences, curated literature data for general PHI information and PHI networks, and PHI-related gene expression data. PHIDIAS currently targets 42 pathogens. These include most category A, B, and C priority pathogens identified by the National Institute of Allergy and Infectious Diseases (NIAID) and the Centers for Disease Control and Prevention (CDC) in the USA, and other pathogens deemed of high priority with regards to public health, such as the human immunodeficiency virus (HIV) and *Plasmodium falciparum *(Table 1).

**Table 1:** Forty-two pathogens included in PHIDIAS

Pathogens (disease)		CDC/NIAID category	No. of genomes	Phinfo	Pacodom	Phinet
1	*Bacillus anthracis *(anthrax)	A/A	3	√	4,588	√
2	*Brucella *spp. (brucellosis)	B/B	4	√	4,267	√
3	*Burkholderia mallei *(glanders)	B/B	1	√	4,679	√
4	*Burkholderia pseudomallei *(Melioidosis)	B/B	2	√	5,093	
5	*Campylobacter jejuni *(food safety threat)	/B	2		3,235	
6	*Clostridium botulinum *(botulism)	A/A	0	√	N/A	√
7	*Clostridium perfringens *(epsilon toxin)	B/B	1		3,770	
8	*Coxiella burnetii *(Q fever)	B/B	1	√	3,032	√
9	*Escherichia coli *(food safety threat)	B/B	6	√	5,440	√
10	*Francisella tularensis *(tularemia)	A/A	2	√	3,057	√
11	*Helicobacter *spp. (gastric ulcer)		5		3,374	
12	*Legionella pneumophila *(legionnaires' disease)		3		3,974	
13	*Listeria monocytogenes *(food safety threat)	/B	2		3,999	
14	*Mycobacterium tuberculosis *(tuberculosis)	/C	2	√	3,991	
15	*Rickettsia prowazekii *(typhus fever)	/C	1	√	2,129	√
16	*Rickettsia rickettsii *(Rocky Mountain spotted fever)	/C	0	√	N/A	√
17	*Salmonella enterica *(food safety threat)	B/B	4	√	5,150	√
18	*Shigella *spp. (food safety threat)	B/B	5	√	5,211	√
19	*Vibrio *spp. (water safety threat)	B/B	5		5,449	
20	*Yersinia pestis *(plague)	A/A	5	√	4,828	√
21	Crimean-Congo hemorrhagic fever virus (tickborne hemorrhagic fever)	C/C	1	√	4	√
22	Eastern equine encephalitis virus (encephalitis)	B/B	0	√	N/A	√
23	Foot-and-mouth disease virus (foot-and-mouth disease)		7	√	3	
24	Guanarito virus (viral hemorrhagic fever)	A/A	1	√	0	√
25	Human immunodeficiency virus (AIDS)		2	√	8	
26	Junin virus (viral hemorrhagic fever)	A/A	1	√	0	√
27	Lassa virus (viral hemorrhagic fever)	A/A	1	√	0	√
28	Louping ill virus (encephalomyelitis)		1	√	6	√
29	Machupo virus (viral hemorrhagic fever)	A/A	1	√	0	√
30	Marburg virus (viral hemorrhagic fever)	A/A	1	√	N/A	√
31	Measles virus (measles)		1	√	0	√
32	Newcastle Disease Virus (Newcastle disease)		0	√	N/A	
33	Powassan virus (encephalitis)		0	√	N/A	√
34	Reston ebola virus (viral hemorrhagic fever)	A/A	1	√	1	√
35	Rift Valley fever virus (Rift Valley fever)	/A	1	√	3	√
36	Variola virus (smallpox)	A/A	2	√	129	
37	Venezuelan equine encephalitis virus (viral encephalitis)	B/B	1	√	8	√
38	Yellow fever virus (yellow fever)	/C	1	√	5	√
39	*Cryptosporidium parvum *(cryptosporidiosis)	B/B	0	√	N/A	
40	*Coccidioides immitis *(meningitis)		0	√	N/A	
41	*Phakopsora pachyrhizi *(soybean rust)		0	√	N/A	√
42	*Plasmodium falciparum *(malaria)		0	√	N/A	
	Total (42 pathogens)		77	37	75,433	27

The program includes 20 bacteria (54 genomes), 18 viruses (23 genomes), and 4 parasites. The database contains 75,433 conserved domains (7,919 unique PSSMs) and PHI network information for 27 pathogens.

## System design

PHIDIAS is implemented using a three-tier architecture built on two Dell Poweredge 2580 servers that run the Redhat Linux operating system (Redhat Enterprise Linux ES 4). Users can submit database or analysis queries through the web. These queries are then processed using PHP/Perl/SQL (middle-tier, application server based on Apache) against a MySQL (version 5.0) relational database (back-end, database server). The result of each query is then presented to the user in the web browser. Two servers are scheduled to regularly backup each others' data.

**Figure 1:**
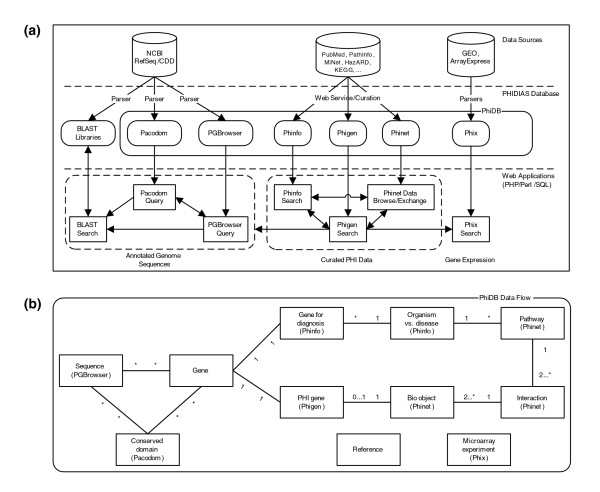
PHIDIAS data flow. **(a) **The PHIDIAS system architecture. **(b) **PhiDB data flow among key elements of different PhiDB database modules. The relationships among these elements are represented by the following signs: *, zero or more; 1, one; and 2...*, two or more. For example, the labeling of a pathway with '1' and '2...*' indicates that one pathway includes two or more interactions.

PHIDIAS includes six components that search and analyze annotated genome sequences, curated PHI data, and PHI-related gene expression data (Figure 1a). Pathogen genomes are displayed and analyzed by PGBrowser, Pacodom, and BLAST searches. The PGBrowser has been developed to browse and analyze the gene and protein sequences of 77 genomes from 42 bacterial, viral, and parasitic pathogens (Table 1). Although PHDIAS does not include non-pathogenic species, PHIDIAS includes genomes from both pathogenic strains (for example, *Escherichia coli *O157:H7 strain Sakai) and non-pathogenic strains (for example, *E. coli *strain K12) in the same pathogen species. Pacodom is used to search and analyze conserved protein domains of the pathogen genomes. Customized BLAST programs allow users to perform similarity searches on pathogen genome sequences. Curated PHI data are separated into Phinfo, Phigen and Phinet, based on general PHI information, PHI molecules and networks, respectively. PHI gene expression experiments and gene profiles are searched through the Phix database system.

PhiDB is the PHIDIAS relational database that integrates different PHIDIAS components. Figure 1b illustrates the relationship and data flow among different database modules and PHIDIAS components. PhiDB integrates PHI-related data from more than 20 public databases (Table 2) and from data curated by the PHIDIAS curation team. PhiDB contains gene information, including sequences, conserved domains from pathogen genomes as well as gene information for PHI and diagnosis of pathogen infections. The biological objects (Bio Object) in the data flow diagram are flexible, that is, they can be a gene or gene product, or any other molecular or cellular entity, including metabolites, cell membrane, mitochondria and so on. The Bio Object element also enables representation of a cluster or group of molecules such as virulent factors and protective antigens. Each interaction includes two or more Bio Objects that function as input or output objects. Each pathway contains more than one interaction. General information pertaining to each pathogenic organism and each disease is available and integrates with pathway and gene information. PHI-related gene expression experiments are also recorded. Detailed information for references, including peer-reviewed journal publications, reliable websites and databases for each of the components is also stored. Each of the PHIDIAS components focuses on different PhiDB elements. All of these components are integrated together and readily available for biomedical researchers working on different pathogens and PHI systems.

**Table 2:** Public databases and software programs integrated in PHIDIAS

Resources	Databases and analysis programs	Comments
**Databases**		
NCBI	RefSeq	Reference sequences
	Genome	Genome summary
	Gene	Gene information
	Protein	Protein information
	Nucleotide	Nucleotide information
	CDD	Conserved domains
	COGs	Clusters of orthologous groups
	Taxonomy	*Brucella *taxonomy information
	PubMed	Biomedical publications
	GEO	Gene expression database
EBI and SIB	ArrayExpress	Gene expression database
	Swissprot	Annotated protein data
	TrEMBL	Protein data
	InterPro	Protein families, domains and functions
	PROSITE	Protein families and domains
VBI	PathInfo	PIML documents via web service
	MiNet	MiNetML documents via web service
TIGR	CMR	Comprehensive microbial resource
	TIGRfam	TIGRfam assignments
	GO	Gene ontology
	KEGG	Pathways
	BioCyc	Biological pathways
	PFam	Protein domains and families
	ProDom	Protein domain families
	PDB	Protein database
University of Michigan	BBP	*Brucella *bioinformatics portal
		
	HazARD	Hazards in animal research database
**Software programs integrated**		
NCBI	BLAST	Blastn, blastp, blastx, tblastn, tblastx, PSI/PHI Blast, Mega Blast, Blast 2 sequences
GMOD	GBrowse	Genome browsing and analysis
	BioPerl	Programming tools
	BioPAX	Biological pathway data exchange format

CMR, TIGR Comprehensive Microbial Resource; GO, Gene Ontology; MeSH, Medical Subject Headings; PDB, Protein Data Bank.

To illustrate the features of data integration and comparative analyses using PHIDIAS, the pathogenic *Brucella *serves as an example and demonstrates how PHIDIAS can promote *Brucella *research. *Brucella *species are Gram-negative, facultative intracellular bacteria that cause brucellosis in humans and animals [17]. *B. melitensis*, *B. suis*, *B. abortus*, and *B. canis *are human pathogens in decreasing order of severity. *Brucella *species have been identified as priority agents amenable for use in biological warfare and bioterrorism and are listed as USA NIAID category B priority pathogens. The genomes of *B. melitensis *strain 16 M [18], *B. suis *strain 1330 [19], and *B. abortus *strain 994-1 [20] and strain 2308 [21] have been sequenced and published.

## PHIDIAS components

### PGBrowser: pathogen genome browser

Pathogen genomes serve as the foundation for the study of PHI in the post-genomic era. PGBrowser integrates data from more than 20 different sources, including NCBI, EBI, and The Institute for Genomic Research (TIGR) (Table 2). Currently, PGBrowser stores 77 genome sequences and 203,297 features from 42 pathogens. NCBI Entrez Programming Utilities are used to download genome information for the pathogens selected from Reference Sequences (RefSeq) and other NCBI databases. The information obtained is formatted in XML. A script has been developed to parse all the protein/gene features, including raw sequences. These are stored in the PhiDB database. Another script has also been developed to query UniProt and other EBI databases, and to download all of the protein information that relates to the 42 pathogens using the SwissProt format. The information is then parsed and stored in a database based on Locus Tag matches. The molecular weights and isoelectric points (pI) are calculated from the protein sequences using the modules (Bio::Tools::pICalculator and Bio::Tools::SeqStats) from BioPerl [22]. In order to enhance the query process, all pathogen sequences and annotation information for PGBrowser are stored in the database server instead of flat files.

**Figure 2:**
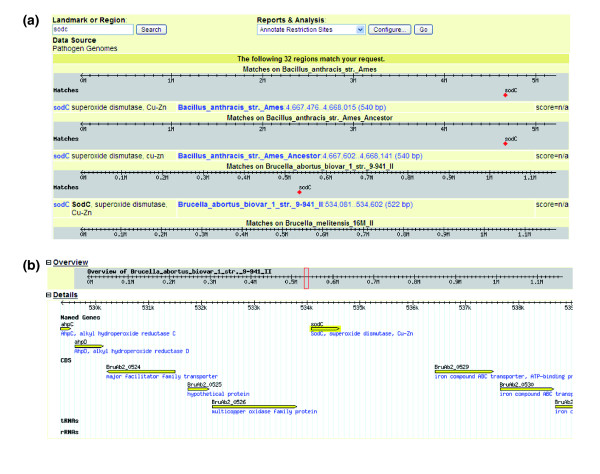
Comparison and analyses of *sodC *genes in the PGBrowser. Thirty two *sodC *genes are found in 32 genomes from 11 bacteria species **(a)**, including sodC from *B. abortus *strain 9-941 **(b)**.

The genome browser web interface of PGBrowser was developed based on the Generic Genome Browser (GBrowse) available at the Generic Software Components for Model Organism Databases (GMOD), a popular genome browser tool because of its portability, simple installation, convenient data input and easy integration with other software programs [23]. The GBrowse program has been used to display genome information about the bacterial pathogens *Brucella *spp. [2] and *Pseudomonas aeruginosa *[24]. PGBrowser modifies GBrowse and allows simultaneous query and analysis for any bacterial or viral gene across all 77 genomes of the 42 pathogens. For example, a query for *sodC *in PGBrowser results in 32 *sodC *hits from 32 genomes in 11 bacterial species, among which are four *Brucella sodC *genes from four *Brucella *genomes (Figure 2a). One can query any *Brucella *gene (for example, *sodC*) among the different *Brucella *genomes, analyze the gene sequences before and after a particular gene (Figure 2b), and obtain gene DNA, RNA, and protein sequences, and perform sequence analyses (for example, finding restriction enzyme digestion sites). As a feature inherited from GBrowse, PGBrowser also provides means for annotating restriction sites, finding short oligonucleotides, and downloading protein or DNA sequence files. PGBrowser can also be directly accessed from other PHIDIAS components such as Pacodom.

**Figure 3:**
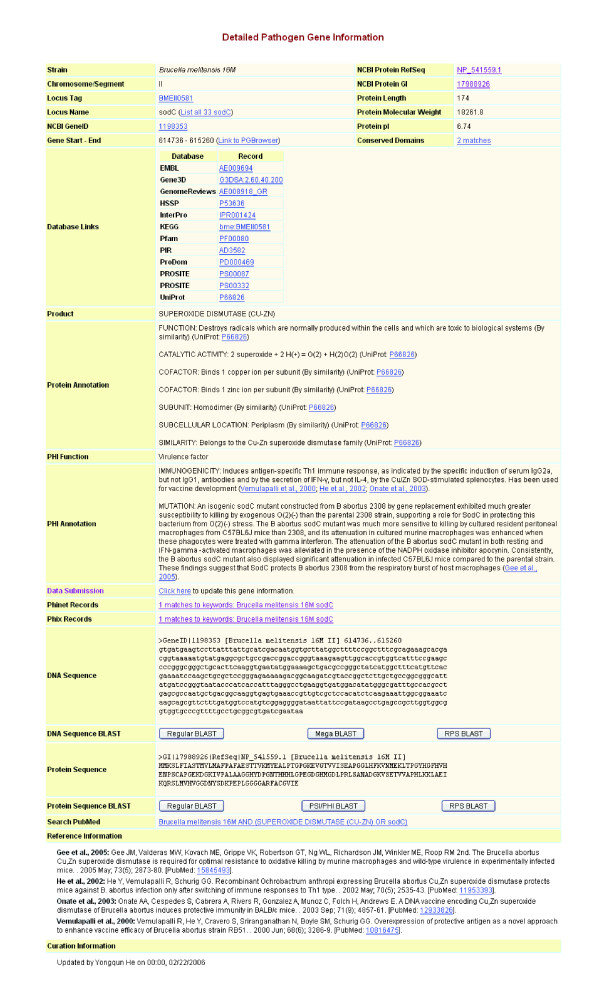
Integrative pathogen gene information in PHIDIAS.

A detailed page of pathogen gene information has been developed to summarize integrative information about a specific pathogen gene, such as *sodC *in *B. melitensis *strain 16 M (Figure 3). It not only provides web links to various databases but also lists detailed protein annotation from authorized databases (for example, UniProt). Additionally, this page includes PHI specific information curated internally by the PHIDIAS curation team. A curator is also prompted to provide additional information using an online submission system. This page also provides DNA and protein sequences in FASTA format. The sequences can be directly linked to a customized BLAST search to find similar sequences from other pathogens. The references for curated PHI information are listed. A PubMed link is available for searching more related peer-reviewed articles. Figure 3 shows that Cu/Zn superoxide dismutase (SOD) encoded by the *B. abortus sodC *gene is required for *Brucella *protection from endogenous superoxide stress [25]. The *B. abortus sodC *mutant is attenuated in macrophages and mice [25]. Figure 3 also indicates that *Brucella *Cu/Zn SOD induces protective Th1 type immune responses and has been used for *Brucella *vaccine development [26]. For comparative purposes, one may examine *sodC *genes from other bacterial pathogens, such as *Bacillus anthracis*. Passalacqua *et al*. [27] recently showed that *B. anthracis *Cu/Zn SOD plays only a trivial role in protecting against endogenous superoxide stress. This indicates that the same gene may have different roles in microbial pathogenesis, suggesting that it is important to analyze pathogen genes individually, particularly in terms of the interactions between pathogens and hosts.

While PHIDIAS is pathogen-oriented and focuses on functional analysis of pathogen genes during PHI, host genome sequences may be requested for gene level PHI analyses. Since GBrowse-based human and mouse genome browsers are publicly available, PGBrowser contains a web interface that allows users to conveniently search the host genome sequence browsers by linking them to the websites.

### Pacodom: pathogen protein conserved domains

The conserved domain data from completely sequenced pathogenic organisms provide valuable information for the identification of protein functions and for the study of PHI. Currently, the NCBI CDD database contains 12,589 position-specific score matrix (PSSM) models that are commonly used representations of motifs present in biological sequences. However, the PSSM models cover a broad range of organisms and, therefore, it is difficult to compare conserved domains from select priority pathogens. To circumvent this problem, a pathogen-specific protein conserved domains database module called Pacodom was developed. This program contains all possible conserved domains found in the 77 pathogen genomes of 42 pathogens. To build this system, a local reverse-position-specific (RPS) CDD library was constructed based on the CDD conserved domain data downloaded from NCBI [28]. The RPS BLAST program (downloaded from the NCBI toolkit distribution) [29] was run for each protein sequence against the RPS CDD library with an expectation value of 10^-6^. The domain alignments obtained from the RPS BLAST search are used to calculate the PSSM. A Perl script was developed to store non-redundant PSSM models [30] in the Pacodom MySQL database module. Currently, the Pacodom database contains 7,919 PSSMs found in 151,787 protein sequences. This value comprises 76.4% of a total of 198,696 proteins from all genomes available in PhiDB.

**Figure 4:**
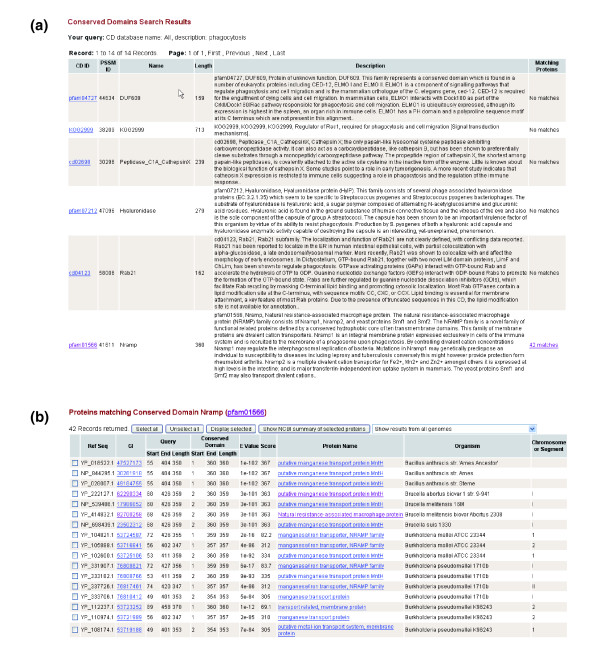
Example of Pacodom applications. **(a) **Pacodom search of 'phagocytosis'. **(b) **There are 42 Nramp protein matches from 42 pathogen genomes of 15 microbial species available in Pacodom.

The conserved domain data from completely sequenced pathogenic organisms provide valuable information for comparative analysis of functional roles of pathogen proteins and their involvement in the interactions between host and microbial organisms. For example, conserved domain data can be used to study phagocytosis, a process where host phygocytic cells (for example, macrophages) engulf pathogen cells (for example, *Brucella*). A search for 'phagocytosis' in Pacodom yields 14 domains; 13 domains do not match any protein from any PhiDB pathogen genome (Figure 4a). However, one domain, 'Nramp' (pfam01566), matches 42 pathogen proteins (Figure 4b). As summarized in the Pfam description of this domain (available in Pacodom), the natural resistance-associated macrophage protein (Nramp) family consists of Nramp1 and Nramp2 in human and mouse systems. Nramp1 plays an important role in phagocytosis and the macrophage activation pathway and regulates the interphagosomal replication of bacteria. Nramp2 is a transporter of multiple divalent cations (for example, Fe^2+^, Mn^2+ ^and Zn^2+^) and is involved in a major transferrin-independent iron uptake system in mammals. The Pfam summary does not list any related microbial Nramp proteins. However, a Pacodom search shows Nramp is very common in the bacterial pathogens listed in PHIDIAS. Those 42 proteins containing the Nramp domain come from many bacterial species, such as *Brucella *spp., *Mycobacterium tuberculosis*, and *Salmonella enterica*. Nramp exists in all strains from these bacteria, whether the strain is pathogenic or non-pathogenic. In contrast, Nramp does not exist in the following species: *Campylobacter jejuni*, *Clostridium perfringens*, *Coxiella burnetii*, *Francisella tularensis*, and *Rickettsia prowazekii*. The Nramp domain has been investigated in depth in mycobacteria [31]. Since pathogenic mycobacteria survive within phagosomes, a nutrient-restricted environment, divalent cation transporters of the Nramp family in phagosomes and mycobacteria may compete for metals that are crucial for bacterial survival [31]. However, inactivation of mycobacterial Nramp, called Mramp, does not affect virulence in mice, suggesting a sufficient redundancy in the cation acquisition systems [32]. A more recent report [33] demonstrated that the *Salmonella enterica *serovar typhimurium (*S. typhimurium*) requires both of the divalent cation transport systems, MntH (Nramp1 homolog) and SitABCD (putative ABC iron and/or manganese transporter), for full virulence in congenic Nramp1-expressing mice. These results suggest that bacterial Nramp is required for pathogenesis in *S. typhimurium *and probably other bacteria by synchronizing with other redundant cation transport system(s) to compete for divalent cations with host cells. The role of *Brucella *Nramp in pathogenesis remains unclear and deserves further analysis. This example demonstrates how Pacadom can be used to find valuable information and form testable hypotheses by comparative analysis of conserved domains.

It is noted that the Nramp domain (pfam01566), while found in a list of pathogens in Pacodom, is also found in many bacterial species that are not pathogens. Therefore, it may be important for investigators to cross reference PHIDIAS search results against databases that contain both pathogen and non-pathogen species. Since Pacodom includes conserved domains from both pathogenic strains and non-pathogenic strains of the same microbial species, it can be used to find domains shown in pathogenic but not in non-pathogenic strains. For example, a query of 'bacteriophage' in Pacodom results in many conserved domains being found, such as Phage_Mu_Gp45 (pfam06890) and Phage_Mu_F (pfam04233), which exist in pathogenic *E. coli *O157:H7 strain Sakai but not in the benign K12 strain. Such domains have previously been reported as required for pathogenesis [34].

### BLAST searches

Gene or protein sequences among different pathogen genomes can be analyzed by different BLAST search approaches. PHIDIAS BLAST uses the latest web server version of BLAST obtained from NCBI [35]. It includes regular BLAST services (blastn, blastp, blastx, tblastn, tblastx), PSI/PHI BLAST, Mega BLAST, RPS BLAST, and BLAST 2 sequences. The nucleotide and protein BLAST libraries contain sequences from all the 77 genomes of the 42 pathogens (Table 1). The 7,919 PSSMs available in Pacodom are combined to form a customized RPS BLAST library specifically used for the RPS BLAST program. The sequence libraries are updated periodically to reflect newly curated annotations and the addition of new genomes.

The approaches used with BLAST greatly help comparative studies for all the genes available in PhiDB. However, some gene annotations from certain genomes are not satisfactory. Based on sequence similarity, these are readily detected with BLAST. The PHIDIAS BLAST methods can also be used to find a group of pathogen genes using a seeding DNA or protein sequence. For example, a PHIDIAS blastp search for the protein sequence of human Nramp1 (also known as SLC11A1, RefSeq#: NP_000569) yields 65 hits from 77 pathogen genomes, most of which are attributable to a single putative manganese transport protein (MntH, which belongs to the Nramp family) found in different pathogens, including four *Brucella *strains. A blastp search using human Nramp2 (also known as SLC11A2, RefSeq#: NP_000608) as input yields similar hits. The BLAST search results are consistent with the analysis of conserved domains as described in the section on Pacodom above.

### Phinfo: curated pathogen-host interaction general information

The Phinfo database module stores pathogen and PHI information curated from the biomedical literature and other curated databases. A major source of Phinfo data are PIML documents available from Virginia Bioinformatics Institute (VBI) [7]. A Java program was developed to extract PIML documents from the ToolBus/PathPort PIML XML database via the PathInfo web service [36]. An Extensible Stylesheet Language for Transformations (XSLT) script was developed to parse the PIML documents into a text-based SQL script. This in turn was used to insert the parsed data into a pre-designed MySQL database system. Phinfo also integrates data manually curated by the PHIDIAS curation team from PubMed literature and other databases such as KEGG [9]. Phinfo links to the Hazards in Animal Research Database (HazARD). This database was developed internally at the University of Michigan [8]. Pathobiology and management of laboratory animals administered USA NIAID/CDC priority pathogens are subjects of the HazARD database and can be searched with Phinfo [8]. Currently, Phinfo includes information for 36 pathogens and corresponding PHI information supported by 2,894 references.

**Figure 5:**
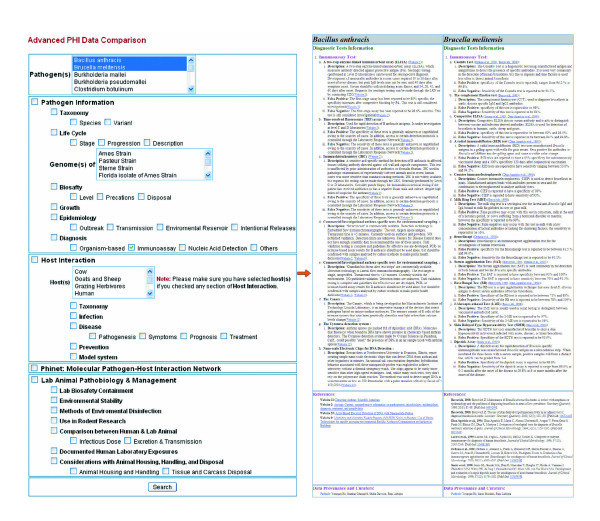
PhiDB Topic Search. The PhiDB Topic Search web interface is shown on the left and a comparison of immunoassays for diagnosis of *B. melitensis *and *B. anthracis *is shown on the right.

Phinfo provides an integrative web interface for user-friendly querying and display of curated pathogen and PHI information. Two query programs are available in Phinfo: Keyword Search and Topic Search. The Keyword Search program allows queries for specific pathogen and PHI information. Such information is displayed with the searched keywords highlighted in color. The Topic Search program searches for one or many of 47 topics listed in the hierarchical structure (Figure 5). Compared to the native PIML XML database [7], the relational Phinfo database system provides secure storage, efficient querying, and database extendibility (that is, the ability to add new data categories). In addition, Phinfo provides links to public databases (for example, NCBI taxonomy, NCBI Gene database, and PubMed). Phinfo is also integrated with other PHIDIAS components. For example, Phinfo of *Brucella *spp. indicates that a PCR assay based on the *B. abortus *gene *wboA *(forward primer: TTAAGCGCTGATGCCATTTCCTTCAC, reverse primer: GCCAACCAACCCAAATGCTCACAA) has been used to differentiate *B. abortus *vaccine strain RB51 from other *Brucella *strains. Either of the primer sequences can be linked directly by clicking to local nucleotide BLAST analysis. Genes found from local BLAST searches are also linked to the PHIDIAS gene table (Figure 3). The *wboA *genes from four *Brucella *genomes are always the first four hits. Other microbial genes (for example, from *Vibrio *and *Yersinia*) are also found, indicating a possible cross-reaction during PCR assays and/or functional similarities among these genes.

### Phigen: pathogen-host interaction genes

The interactions between pathogen and host genes have been extensively studied in the post-genomic era [37]. However, most databases of genes and proteins focus on sequence annotation and function in a single cell species. Phigen focuses on functional annotation of pathogen genes and their interaction with host genes during the process of pathogen-host reactions. The main source of the PHI-related gene annotation comes from literature curation and data integration. The information about genes and/or proteins required for virulence, able to induce protective immune responses in hosts, or used for diagnosis, has been annotated and stored in the Phigen system. Phigen consists of two parts, pathogen gene search and manual curation submission.

**Figure 6:**
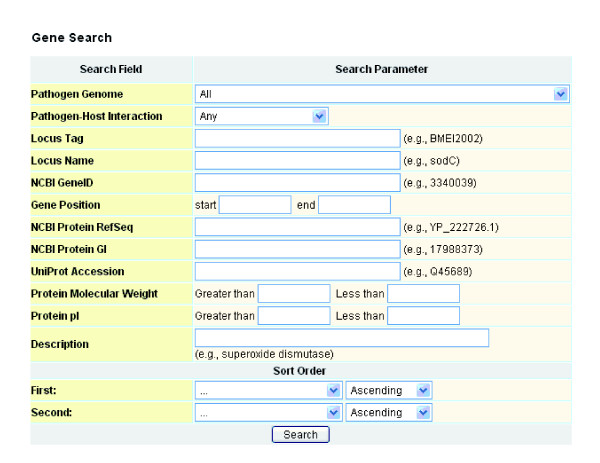
Gene search web interface in Phigen.

Every pathogen gene may be involved in an interaction between the pathogen and its host. The pathogen gene search interface of Phigen allows users to search for any pathogen genes from the 77 genomes of the 42 pathogens available in PhiDB (Table 1). The Phigen search has a function for simple Boolean-powered keyword searches and an advanced topic search (Figure 6). The advanced topic search allows searching for PHI-specific information and generic features, including chromosomes and chromosomal position, RefSeq identifier, GenBank accession number, locus tag and name, molecular weight, pI, and description. Searched results can also be sorted in ascending or descending order. Molecular weight and pI data obtained in each search may be used to aid the interpretation of two-dimensional mass spectrometry data for proteomics analyses.

Phigen provides an efficient online submission system for submitting of data for curation of pathogen genes, especially their roles in PHI. The information is fully referenced from peer-reviewed publications, with direct links to PubMed paper abstracts and full texts for additional details. Submitted information is critically reviewed and verified by reviewers prior to acceptance. Currently, Phigen has manually curated and stored more than 400 genes from 42 pathogens. Instead of altering records from other public databases, the curation is currently focusing on adding PHI-related information, such as host immune responses, gene mutations and resultant pathogenic changes in the host. In addition to integrated gene information, the PHI-specific information assists researchers in surveying, comparing, and studying gene-specific PHI mechanisms.

### Phinet: pathogen-host interaction network curation, data exchange, and visualization

PHI has the ability to reveal complicated networks between pathogen and host molecules. Phinet is targeted at analyzing molecular networks responsible for PHI. Phinet data are stored in PhiDB and are derived from the MINetML XML database extracted through the web service, other curated databases (for example, KEGG), and manual annotation based on literature curation. Similar to that implemented in Phinfo, a Java program was developed to extract MINetML documents from the ToolBus/PathPort MINetML XML database via the MINet web service [38]. An XSLT script was further developed to parse the MINetML documents into a text-based SQL script, which is used to insert the parsed data into a pre-designed MySQL database system. Data from the KEGG pathway database are manually curated and added to Phinet. Phinet also includes a web-based data submission system that permits internal or external curators to submit PHI-related network data. The Phinet data submission follows a similar curation policy as described for Phigen online submission above. If conflicts exist for data from different sources, those records with the strongest reference support are selected, or in some circumstances, conflicting data were included with well-documented references. Currently, Phinet includes PHI network information for 21 pathogens.

**Figure 7:**
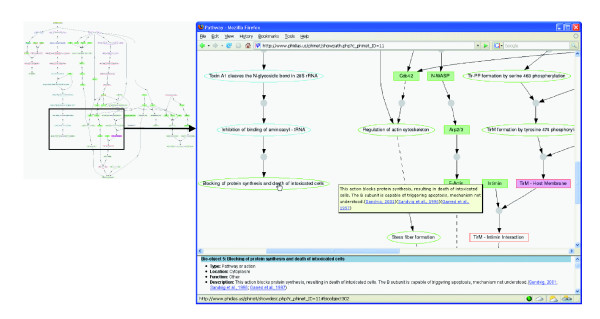
Visualization of an *E. coli *pathogenesis network in Phinet. A click on each node provides detailed information about a biological object in the bottom frame. When a mouse cursor moves over a node, a brief description of the biological object will appear. An interaction between biological objects is represented by a centered gray ball and arrows between nodes. Once the centered gray ball is clicked, details about the specific interaction appear in the bottom frame. Subcellular locations of biological objects are differentiated by the node border colors. The biological object types (for example, protein or gene) are represented by a combination of the node background colors and shapes. The program also displays different interactions, such as inhibition (solid T sign), activation (solid arrow), and indirect effects (dashed line).

A Graphviz-based visualization software program has been developed internally to dynamically display all the biological interactions in Phinet (Figure 7). The visualization program effectively displays all pathway data for each pathogen available in Phinet. The user can select to view information about a biological object or the interaction between biological objects (Figure 7).

Data exchange among different pathway databases is critical for data sharing and integration. BioPAX is a community-supported data exchange format for biological pathway data [14]. Current BioPAX Level 2 covers metabolic pathways, molecular interactions and protein post-translational modifications. Compared to the model representation format SBML, BioPAX focuses on molecule and interaction classification schemes and database cross-referencing for pathway components. PHI networks involve complex signaling pathways and gene regulatory networks that are similar to BioPAX, although they are not supported in their entirety by the current BioPAX version. A program was developed to transform Phinet data to the closest BioPAX OWL format using the current BioPAX Level 2 format. These BioPAX documents can be used to communicate with other biological pathway databases and, additionally, provide input files for other software programs.

### Phix: pathogen-host interaction gene expression

Gene expression data for pathogens and/or hosts during PHIs comprise important data for analysis of pathogen pathogenesis and host defense mechanisms. The NCBI GEO [15] and EBI ArrayExpress [39] are the two biggest repositories that store publicly available microarray and proteomics data, many of which relate to PHI. The Phix database stores all gene expression experiment records for the targeted 42 pathogens and their infected hosts from the GEO and ArrayExpress databases. Since new gene expression experiments are frequently submitted to these databases, a Linux cron job [40] was developed to check daily for any new information; if found, the new data are added to the database. The Phix module currently stores 187 GEO records and 79 ArrayExpress records. The Phix gene expression search program provides a one-step system for users to query PHI gene expression experimental data. For example, a query of 'macrophage' in Phix leads to 13 search hits representing various experimental studies involving pathogen-infected macrophages. Each hit links to detailed information in GEO or ArrayExpress. These results are particularly useful for comparing different pathogen-macrophage interaction systems. Finally, Phix also includes a gene profile search engine for query and comparison of expression profiles of specific genes from one, or all, of the pathogen genomes selected from the GEO and ArrayExpress databases. In contrast to the general GEO and ArrayExpress gene profile search engines, this program is specifically targeted to pathogen and PHI studies.

To improve further integration of different PHIDIAS components, the PHIDIAS web site contains a keyword search engine that simultaneously allows searching for information from all PHIDIAS components. All results are sorted based on the components and displayed in one page for convenient data analysis (data not shown).

## Discussion

A deeper understanding of PHI is required for effectively combating infectious diseases. To efficiently analyze the ever-increasing amount of PHI data in the post-genomics era, PHIDIAS was developed. This program permits integration of PHI related data from genome sequences, the biomedical literature, curated databases, and gene expression experiments. PHIDIAS covers 42 microbial and viral pathogens of high priority for public heath and security. The gene and protein sequences from each genome are available for browsing and analysis using PGBrowser and customized BLAST searches. The conserved domains are analyzed and stored in Pacadom. PHI data extracted from existing databases, or internally manually curated, are stored in Phinfo (general PHI information), Phigen (PHI genes) and Phinet (PHI networks). PHI-related gene expression experiment records and profiles from public GEO and ArrayExpress repositories can be directly searched in Phix. The PHIDIAS components are interconnected (Figure 1). Scenarios have been used in this report to show that PHIDIAS greatly helps *Brucella *research by allowing users to search and analyze integrative *Brucella *data derived from different sources and compare these data with those from other pathogens.

Similar PHI-related biological programs exist. PHI-base is a web-accessible database devoted to the identification and presentation of information on fungal and oomycete pathogenicity genes and their host interactions [41]. PathoPlant deals with plant-pathogen interactions, signal transduction reactions, and microarray gene expression data from *Arabidopsis thaliana *subjected to pathogen infection and elicitor treatment [42]. In contrast to PHI-base and PathoPlant, which target the interactive relationships between pathogens and hosts, PHIDIAS includes a list of other bacterial, viral and parasitic pathogens and their interactions with hosts. Similar to PHIDIAS, PHI-base and PathoPlant contain manually curated information supported by strong experimental evidence (gene disruption experiments) and literature references. Each system allows interlinking of gene information with external data sources. However, PHIDIAS integrates more data sources for a broader scope of data integration and analysis. PHIDIAS also provides on-line submission systems for curators to submit annotated data for genes as well as genetic interactions and pathways.

Many biological systems allow systematic genome comparison. MicrobesOnline is a publicly available suite of web-based comparative genomic tools designed to facilitate multispecies comparison among prokaryotes [43]. The database PRODORIC systematically organizes information about the prokaryotic gene expression of multiple prokaryotic species, and integrates this information into regulatory networks [44]. As does PHIDIAS, these systems contain many comparative analysis and visualization tools. However, while MicrobesOnline and PRODORIC target more general prokaryotic species, PHIDIAS focuses on pathogenic bacteria as well as viral and parasitic pathogens important for biodefense and/or human health. PHIDIAS also emphasizes interactions between pathogens and hosts, which MicrobesOnline and PRODORIC currently lack. PHIDIAS also contains manually curated data for functional annotation of genes and genetic networks in pathogen genomes.

Eight Bioinformatics Resource Centers (BRCs), sponsored by the USA NIAID, provide web-based resources for organisms that are considered potential agents of biowarfare or bioterrorism or cause emerging or re-emerging diseases [45]. Each BRC is targeted to maintain and annotate genomes from a selected list of pathogens. Each BRC contains a web site to display the data and analyses for these pathogens. BRC Central [46] serves as a repository linking these eight BRCs. Many of the pathogens contained in the BRCs are also found in PHIDIAS. However, PHIDIAS also targets non-biodefense pathogens (for example, HIV) not included in the BRCs. Additionally, PHIDIAS includes not only data analysis and search functions found in the BRC resources, but also provides tighter integration of various data types. Finally, PHI and literature data curation are emphasized in PHIDIAS but not in the BRCs.

PHIDIAS is unique in that it integrates existing knowledge about a broad range of human or zoonotic priority pathogens, and focuses on efficient searching, visualization, comparison, and analysis of pathogen genes and their interactions with their hosts using genome sequences, manually curated literature data, and gene expression data from public resources. PHIDIAS utilizes online data submission systems for efficient data curation, making integrative PHI data more comprehensive. All the PHIDIAS components are scalable, and more pathogens and PHI systems may be added to the system. Due to inclusion of an ever increasing number of pathogens in PHIDIAS and in view of the dramatically increasing amount of literature information, it will be an ongoing challenge to curate all the significant genes and keep the PHI-related information in PhiDB current. Therefore, one of our future directions will be to explore ontology-based natural language processing and statistical methods for efficient literature acquisition and curation. In this regard, we have now developed a literature mining and curation system (Limix). This system has been used efficiently for literature mining and curation for four *Brucella *genomes [2]. Systematic curation and incorporation of *Brucella*-specific mutation and genetic interaction information has allowed a comprehensive investigation of *Brucella *pathogenesis [2]. Limix is currently being expanded to annotate literature for other pathogens and PHI systems. Finally, future plans for expanding PHIDIAS include development of a web-based database and an analysis pipeline that permit storage, processing, and modeling of PHI-related gene expression data. This approach will allow researchers to address scientific PHI questions with the ultimate goal of successfully fighting infectious diseases.
